# Cost‐effectiveness analysis of dabigatran, rivaroxaban and warfarin in the prevention of stroke in patients with atrial fibrillation in China

**DOI:** 10.1186/s12913-021-06084-1

**Published:** 2021-01-28

**Authors:** Hongtao Wei, Can Cui, Xiangli Cui, Yi Liu, Dandan Li

**Affiliations:** Department of Pharmacy, Beijing Friendship Hospital, Capital Medical University, 100050 Beijing, China

**Keywords:** Warfarin, Cost‐effectiveness, New oral anticoagulants, Chinese population

## Abstract

**Background and objective:**

To evaluate the cost-effectiveness of new anticoagulants and warfarin in the prevention of stroke in Chinese patients with atrial fibrillation (AF).

**Methods:**

The Markov model was constructed to compare patients’ quality-adjusted life-years (QALYs) using drug cost, the cost of the examination after taking a drug, and the incremental cost of other treatments. Both dabigatran (110 and 150 mg, twice a day) and rivaroxaban (20 mg, once a day) were compared with warfarin (3–6 mg, once a day). Willingness to pay, three times the 2018 China GDP per capita (9481.88 $), was the cost-effect threshold in our study.

**Results:**

The total cost were was 5317.31$, 29673.33$, 23615.49$, and 34324.91$ for warfarin, rivaroxaban, dabigatran 110 mg bid, and dabigatran 150 mg bid, respectively. The QALYs for each of the four interventions were 11.07 years, 15.46 years, 12.4 years, and 15 years, respectively. The cost-effectiveness analysis of the three new oral anticoagulants and warfarin showed that the incremental cost-effectiveness ratio (ICER) was 5548.07$/QALY when rivaroxaban was compared with warfarin. Rivaroxaban was the most cost-effective choice and warfarin was the least.

**Conclusions:**

In Chinese patients with AF, although warfarin is cheaper, rivaroxaban has a better cost-effectiveness advantage from an economic point of view.

## Background

Atrial fibrillation (AF) is one of the most common cardiac arrhythmias, Which could increase the incidence of diseases such as acute ischemic stroke, heart failure, and myocardial infarction [1]. The prevalence of AF in China is 0.61 to 0.77 % [2], which was 0.3–0.48 % higher than the average international rate [3, 4]. The total incidence of AF was 1.46 % in southern China during 2017–2018 [5, 6]. The incidence increased with age, reaching 5 % in patients aged 80 years or older [7]. Around 20–30 % of ischemic stroke was caused by AF [8], which might likely lead to disability and significantly reduce the quality of life for patients [9–11] and increase the burden of chronic care [12, 13]. Therefore, effectively to prevent AF-induced cardiogenic embolism is particularly essential.

In the past, warfarin was the most effective method to prevent stroke caused by AF. However, the use of warfarin is complicated, and it needs to be closely monitored with INR values. Moreover, some studies have shown that the preventive effect of warfarin is significantly lower in Asians than Caucasians, and bleeding events are more likely to occur in Asians [14, 15]. In recent years, novel oral anticoagulants (NOACs), which have better safety, efficacy, and compliance properties, provide a new choice for AF patients [16, 17]. However, the NOACs are more expensive. This article aimed to evaluate whether NOACs, dabigatran, and rivaroxaban, have a cost-effectiveness advantage compared with warfarin for stroke prevention among AF patients in China through pharmacoeconomics analysis.

## Methods

### Evaluation method

This study started with the payer and used a cost-effectiveness analysis to construct a Markov model. The model was used to compare the cost and effectiveness of warfarin, rivaroxaban, and dabigatran (110 mg and 150 mg) in the prevention of stroke and myocardium infarction in patients with non-valvular AF (NVAF).

### Model specification

**Fig. 1:**
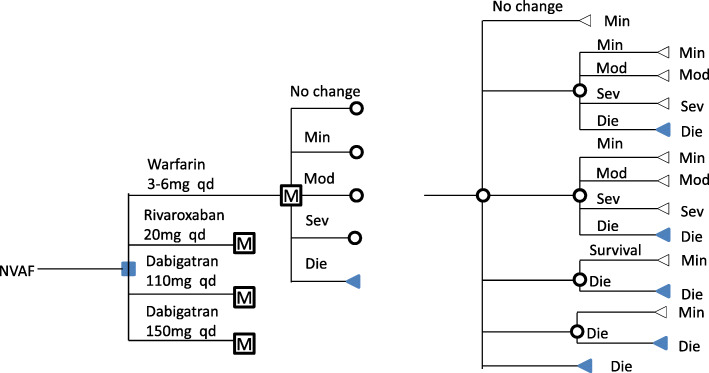
Representation of the Markov model. The four treatment options are shown on the left. “M”represents a Markov process with 4  health states. These health states are identical for each treatment option. “Min” represent state without incident or sequelae, “Mod” represent moderate disabilities, “Sev” represent completely disabilities , Die means death. All patients remain in the “Min”state until one of the five events occurs. NVAF, nonvalvular atrial fibrillation; IS, ischemic stroke; ICH, intracranial hemorrhage; ECH, extracranial hemorrhage; MI, myocardial infarction

An individual-level simulation model was built to predict the clinical events and outcomes of each patient over time under different treatment regimens. According to the natural history of disease development, the survival status of patients with NVAF was divided into four conditions: mild (no event of AF or no sequelae of events), moderate (moderate disability survival), severe (need help to survive) and death. Clinical manifestations in the model simulation were ischemic stroke (IS), intracranial hemorrhage (ICH), extracranial hemorrhage (ECH), myocardial infarction (MI) and death. At the beginning of the cycle, all patients were of mild status. With the cycle running, clinical events occurred, and individuals switched between states. The flow chart of the model is shown in Fig. 1. The model cycle was one year, and the study period was 30 years with a discount rate of 3 %. Willingness to pay, three times the 2018 China GDP per capita (9481.88 $), was the cost-effect threshold in our study .

### Model Assumptions

We assumed the following hypothesis: (1) patients can experience any but only one clinical event in each cycle, (2) the conversion rate of each event in the model does not change with time, (3) assuming extracranial hemorrhage and MI have only two outcomes, namely patients with mild illness or death, (4) severely ill status is a state of irrecoverable disability and entirely in need of survival. It is assumed that once an individual enters the state of a severely ill condition, there are only two outcomes: maintenance and death, and (5) ignoring the occurrence of clinical events may lead to changes in costs due to drug withdrawals and others.

### Sensitivity Analysis

One-way sensitivity analyses were performed using Tree Age Pro 2011 software to test the robustness of the model results. Plausible ranges were obtained from the literature. To evaluate the impact of the uncertainty in all variables simultaneously, a probabilistic sensitivity analysis was performed using the Monte Carlo simulation. Probabilistic sensitivity analyses were performed through the second-order Monte Carlo simulation model (1,000 times). Costs varied after assuming a log- normal distribution. Probabilities and utilities varied according to a beta distribution. Uncertainty was represented on a scatter-plot and cost-effectiveness acceptability curve.

### Data sources

#### Therapeutic effect and conversion rate

Transition probability refers to the probability that a patient moves from one state to various states in one cycle. To obtain the closest conversion rate of the Chinese population, Re-ly15, XANTUS16, ROCKET AF17 trials, and relevant literature were considered18-20. All included subjects were Chinese or subgroup analysis of the Asian population. The Re-ly trial selected 541 AF patients from the Chinese subgroup, which was a randomized efficacy comparison warfarin and dabigatran (110 mg or 150 mg, twice daily) in long-term anticoagulant therapy. The XANTUS trial included 2,273 Chinese (including Hong Kong and Taiwan), which was a prospective real-world observation study, comparing different doses of rivaroxaban in NVAF patients. Results are shown in Table 1.

**Table 1:** Base-case model variables and ranges used in a sensitivity analysis

Variable	Value	Range	Reference
Probabilities			
Probability of ischemic stroke caused by different drugs			
Warfarin	0.04	0.023–0.0453	[18–20]
Rivaroxaban	0.0193	NA	[19]
Dabigatran110mg	0.0183	0.0167–0.0189	[18, 19]
Dabigatran150mg	0.0137	NA	[20]
Probability of ischemic stroke outcome by severity			
Light	0.091	0.091–0.133	[21]
Moderate	0.425	0.348–0.425	[21]
Severe	0.402	0.402–0.417	[21]
Die(in 30day)	0.082	0.082–0.101	[21]
Probability of ICH caused by different drugs			
Warfarin	0.0121	0.0057–0.0294	[18, 19, 22]
Rivaroxaban	0.00257	0.0021–0.0033	[19, 22]
Dabigatran110mg	0.00359	0.0028–0.0039	[18, 19]
Dabigatran150mg	0.0027	NA	[18]
Probability of ICH outcome by severity			
Light	0.12	NA	[21, 23]
Moderate	0.27	NA	[21, 23]
Severe	0.43	NA	[21, 23]
Die	0.18	NA	[21, 23]
Probability of ECH caused by different drugs			
Warfarin	0.027	NA	[24]
Rivaroxaban	0.03	NA	[23]
Dabigatran110mg	0.007	NA	[24]
Dabigatran150mg	0.0217	NA	[23]
Probability of ECH outcome by severity			
Die	0.0147	0.01–0.04	[21]
Probability of MI caused by different drugs			
Warfarin	0.0098	NA	[21]
Rivaroxaban	0.0098	NA	[21]
Dabigatran110mg	0.0072	NA	[18]
Dabigatran150mg	0.0074	NA	[18]
Probability of MI outcome by severity			
Death	0.166	0.158–0.174	[21, 25]
All-cause mortality by different drugs			
Warfarin	0.026	0.0258–0.0261	[18, 22]
Rivaroxaban	0.0164	NA	[22]
Dabigatran110mg	0.0333	NA	[18]
Dabigatran150mg	0.0219	NA	[18]
Cost			
Price (specification/$)		Range of daily dose	
warfarin	3 mg/0.07	1.5 mg-6 mg/d	
Rivaroxaban	20 mg/4.87	15–20 mg qd	
Dabigatran110mg	110 mg/2.34	110–150 mg bid	
Dabigatran150mg	150 mg/3.01	110–150 mg bid	
Cost of examination and service	10.77	NA	
Frequency about examination/Annual			
warfarin	21	NA	[26]
Rivaroxaban and Dabigatran	1	NA	
Total cost $(drug and examination)			
Warfarin	253.30	239.70-280.51	
Rivaroxaban	1787.00	1435.86-2844.52	
Dabigatran110mg	1718.21	1711.17-2206.93	
Dabigatran150mg	2206.93	1711.17-2206.93	
Event			
Stroke	1351.20	851.36-2681.69	
ICH	2605.43	1935.86-3862.05	
ECH	1216.72	867.23-2516.68	
MI	3875.18	1805.02-5529.89	
Health utility values in each state			
Light	0.76	0.7–0.9	[27]
Moderate	0.39	0.1–0.5	[27]
Severe	0.16	0.0-0.32	[27]
ICH	0.8	0.79–0.84	[28]
ECH	0.8	0.79–0.84	[28]
MI	0.84	0.67–0.96	[29]

*ICH* Intracranial hemorrhage; *ECH* Extracranial hemorrhage; *MI* Myocardial infarction

#### Cost

The state of NVAF event-free costs were the average annual direct medical costs of the four treatment measures, including medical service costs, medication costs, and related examination costs. The medical service charge was 50 yuan per time for general outpatient service in the tertiary hospital of Beijing. The drug cost referred to the public price from the Beijing Sunshine Drug Procurement platform. The coagulation function monitoring cost was 3.73 $/test. Warfarin’s coagulation function monitoring frequency was based on the 2015 guidelines for stroke prevention and treatment in patients with AF in China23. INR is monitored 21 times a year, and the monitoring frequency for NOACs is once a year. The average hospital expenses for acute events were obtained from the 2018 China Health Statistics Yearbook, as shown in Table 1.

The dose of warfarin was adjusted based on INR, and the daily dose fluctuated between 1.5 and − 6 mg, resulting in a change in drug costs. The recommended dosage of rivaroxaban for an adult with NVAF was between 15 and 20 mg/d. There are three dosage strengths of 10 mg, 15 mg, and 20 mg for rivaroxaban, and the cost of 20 mg/day of rivaroxaban was used in our analysis. The dosage of dabigatran was adjusted from 110 mg or 150 mg, twice daily, according to the risk of bleeding, as shown in Table 1.

#### Value of health utility

The quality-adjusted life year (QALY) was adopted as the health utility index in this analysis. The value of health utility was derived from a similar population investigation and published literature. Assuming that QALY is 1 for health and 0 for death, EQ-5D was used to calculate the quality of life under specific condition, and the health utility values in each state are shown in Table 1.

## Results

### Basic analysis

.

The results showed that the total cost of warfarin, rivaroxaban, dabigatran (110 mg bid, and 150 mg bid) in NVAF patients was 5317.31 $, 29673.33 $, 23615.48 $, and 34324.91 $, respectively. The available QALY value was 11.07, 15.46, 12.4, and 15 years, respectively. The efficiency of dabigatran 150 mg was the lowest due to its high cost. According to the recommendation of the World Health Organization on pharmaceutical economics evaluation, we took three times of the 2018 China GDP as the cost-effect threshold in our study. The result of an incremental cost-effectiveness ratio (ICER) showed that rivaroxaban was 5548.07 $/QALY, which was the best therapeutic regimen. The results are shown in Table 2.

**Table 2:** cost-effectiveness comparison

	Utility(QALY)	Increased utility	Cost($)	Increased costs	ICER
warfarin	11.07		5317.31		
Rivaroxaban	15.46	4.39	29673.33	24356.02	5548.07
Dabigatran110mg	12.40	1.33	23615.48	18298.17	13758.02
Dabigatran150mg	15.00	3.93	34324.91	29007.60	7381.07

2.2 Sensitivity Analysis.

**Fig. 2:**
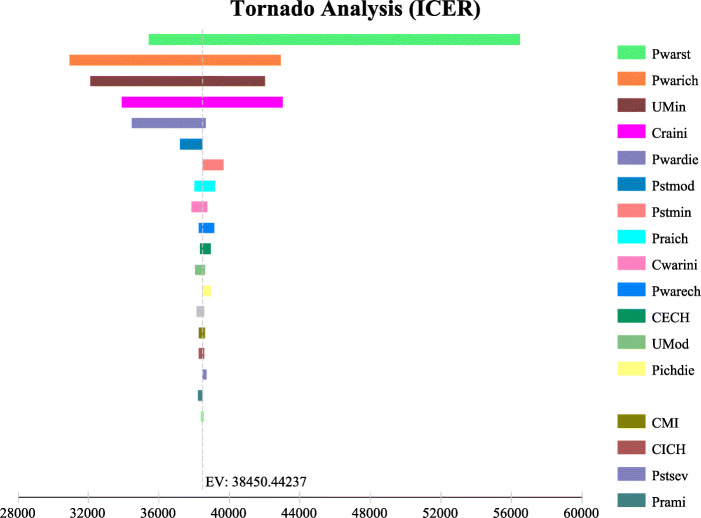
Tornado analysis (ICER). Cost per additional quality-adjusted life year (bars) of rivaroxaban compared to adjusted-dose warfarin as determined in tornado diagram over plausible ranges for all variables. The willingness-to-pay threshold of 202295.67 yuan per quality-adjusted life year are presented.

Tornado analysis showed that PwarST (probability of ischemic stroke in warfarin), PwarICH (probability of hemorrhagic stroke in warfarin), Umin (health utility value of mild patients), and Criv (rivaroxaban price) were the most influential variables of this model, as shown in Fig. 2.

**Table 3:** one-way sensitivity analysis results

ICER(Cost/QALY)	one-way sensitivity analysis
Basic analysis	5548.07
PwarST Adjust to maximum	5112.44
PwarST Adjust to minimum	8176.64
PwarICH Adjust to maximum	4470.03
PwarICH Adjust to minimum	6189.53
Umin Adjust to maximum	4645.53
Umin Adjust to minimum	6058.70
Cost of rivaroxaban Adjust to maximum	9281.03
Cost of rivaroxaban Adjust to minimum	4311.95

*ICER* incremental cost-effectiveness ration; *PwarST* Risk of cerebral infarction with warfarin; *PwarICH* Risk of intracranial hemorrhage with warfarin; *Umin* Quality of life adjusted years in patients with mild dysfunction

A sensitivity analysis was carried out to assess these four significant, influential factors. The results showed that when the probability of ischemic stroke and hemorrhagic stroke in warfarin was adjusted to the minimum, the ICER of rivaroxaban compared to warfarin was 8176.64 and 6189.53 $, respectively. When ICER is less than one time the GDP, rivaroxaban had absolute economic advantages. When the annual quality of life Umin in the mildly diseased state was adjusted to a maximum value of 0.9 and a minimum value of 0.7, the ICER of rivaroxaban over warfarin was 4645.53 $ and 6058.70 $, respectively. Rivaroxaban still had absolute economic advantages. When the price of rivaroxaban was adjusted to the maximum value, the ICER was 9281.07 $, and rivaroxaban still maintained an absolute economic benefit, which was consistent with the baseline analysis, as shown in Table 3.

### Probabilistic sensitivity analyses

**Fig. 3:**
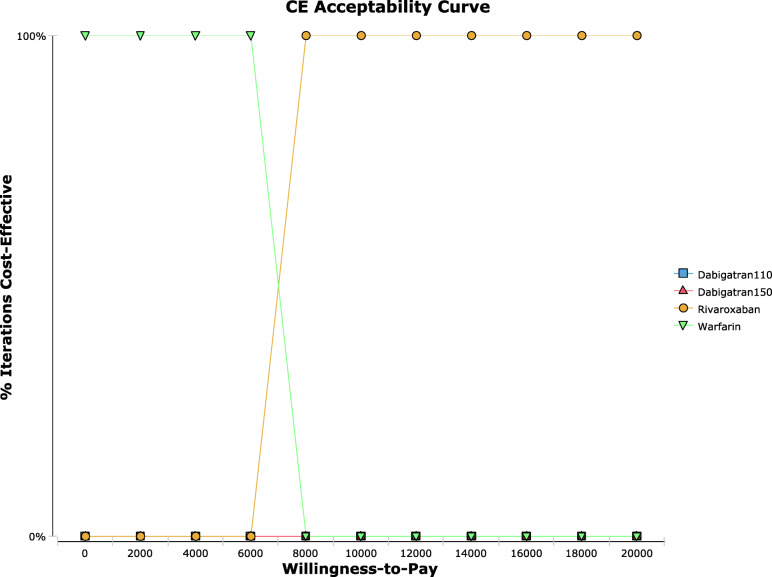
Cost-effectiveness acceptance curve. Cost-effectiveness acceptability curve results based on 1000 Monte Carlo simulations of the model. The curve presents the probability that the rivaroxaban regimen is cost-effective as a function of willingness-to-pay threshold

**Fig. 4:**
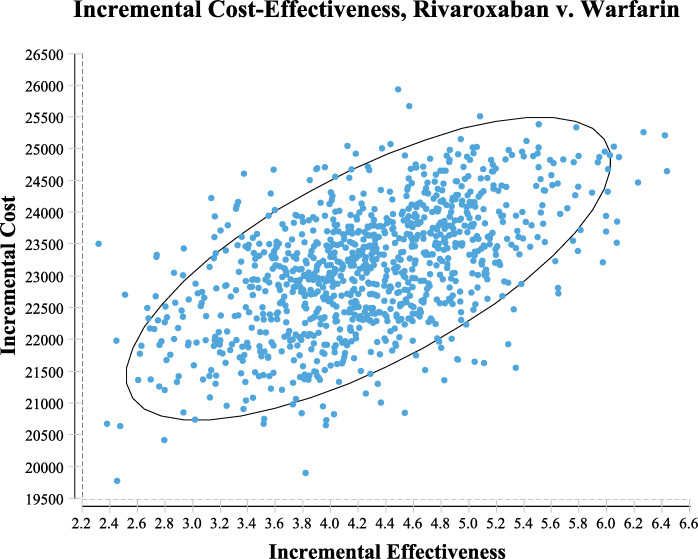
Incremental Cost-Effectiveness Rate scatter. Incremental cost-effectiveness scatterplot of the result of the probabilistic sensitivity analysis. Each point represents a simulation. Ellipse represents 95% confidence interval ellipse

The cost-effectiveness acceptable curve was obtained (Fig. 3). When the WTP was greater than 7587.27 $, the acceptable probability of rivaroxaban was close to 100 %, and dabigatran was always in a disadvantaged scenario. The ICER scatter plot (Fig. 4) reflects the change and concentration of the ICER value in the probabilistic sensitivity analysis. When the WTP was three times the GDP 28445.64 $, most values fell within the confidence interv al. Rivaroxaban was more cost-effective than other options.

## Discussion

In this study, previously published population data of patients with AF in China receiving different oral anticoagulant treatments were included in the Markov model for cost-effectiveness analysis. The weighted average method was utilized in our research. Transition probability data came from randomized controlled trials and the utility values date were from cohort studies or population-based studies. In the real world, many factors [30–32], such as low patient compliance and medication errors, might have influenced disease [33, 34]. Thus, it might be difficult to estimate these factors. Therefore they were not calculated or discussed in our research.

As a common chronic disease, the Markov model is used to simulate the disease’s progression and control, which has certain significance in guiding the long-term clinical use of medications. It has been reported that the age of patients with atrial fibrillation is between 20 and 99 years old [35]. The survival time of patients with atrial fibrillation can be several decades. Therefore we choose a more extended period of 30 years for the cycle simulation.

The cost data of this study includes three aspects: the cost of the drug, the examination fees when taking the drug, and the cost of the treatment after disease occurrence. The cost of drug treatment is based on fixed drug pricing from the Beijing drug procurement platform. The examination fees are the fees set by the medical institution. Both of these two types of fees are state-controlled prices. The cost of treating AF-induced stroke or MI is the average cost listed in the 2018 Chinese Health Yearbook. From the cost of drug treatment alone, the average daily cost of warfarin is 0.04–0.15 $, which is much lower than 4.68 $ for dabigatran 110 mg, 6.02 $ for dabigatran 150 mg, and 4.87 $ for rivaroxaban. However, warfarin needs to monitor the INR regularly for a long time, and each monitoring visit requires consultation and examination fees. After adding these fees, the average treatment cost of warfarin is only 0.39 $, still far lower than dabigatran and rivaroxaban.

The results of this study show that the use of warfarin QALYs is 11.07, and the cost of drug treatment, examination, and disease treatment for 30 years is 5317.31$. Since warfarin has a higher risk of stroke, the cost of treating cardiogenic embolism and subsequent rehabilitation is higher [36, 37], therefore, more effective treatments should be selected. Compared with warfarin, for each additional QALY, the costs are rivaroxaban 5550.18 $, dabigatran (150 mg) 13772.09 $, and dabigatran (110 mg) 7381.07$. In 2017, the per capita GDP of China was 9481.88 $ [38]. We take three times the GDP as WTP for further analysis. Rivaroxaban has the highest cost-effectiveness, followed by warfarin. Dabigatran 150 mg and 110 mg have poor cost-effectiveness. Among them, dabigatran 150 mg has an extended advantage, and dabigatran 110 mg has an absolute disadvantage. When WTP is lower than 53945.51 yuan, warfarin has the highest cost-effectiveness, which is similar to previous studies in Taiwan [39], South Korea [40], and Hong Kong [19].

Previous foreign studies have shown that all NOACs have cost-effectiveness advantages compared to warfarin. Among them, apixaban has the best cost-effectiveness in preventing stroke in patients with atrial fibrillation. However, because of the latest approval of apixaban in mainland China and limited clinical use, apixaban was not included in the analysis. Rivaroxaban and dabigatran are sold at different prices in different regions, leading to changes in cost-effect results. If the price of rivaroxaban is reduced by 30 %, rivaroxaban has a better cost effect. At a willingness-to-pay threshold of £20,000 per quality-adjusted life-year (QALY), all NOACs had the positive expected incremental net benefit (INB) compared with warfarin.

In this study, a single-factor sensitivity analysis was performed using Tree Age Pro 2011 software. With the WTP value of 8452.27 $, tornado plot analysis shows that PwarST (probability of ischemic stroke in warfarin), PwarICH (probability of hemorrhagic stroke in warfarin), Umin (year of quality of life in mildly diseased condition), and Criv (rivaroxaban price) are the most influential parameters for the model. The probability of ischemic stroke with warfarin is the most influential factor in the model.In previous studies, the effective control rate of INR was also an important influencing factor when taking warfarin [41]. When time in therapeutic range ≥ 65 %, the risk of ischemic stroke was reduced by warfarin [19]. However, whether warfarin therapy is well-managed or not,rivaroxaban still has absolute economic benefits. The probability of ischemic stroke in warfarin adjusted from minimum to maximum, the ICER of rivaroxaban is adjusted from 6189.53 $ to 9281.03 $, which is consistent with the analysis of baseline results.

## Limitations

Furthermore, several limitations are worthy of discussion in this study. First, our research mainly focuses on the results of the Chinese population, but the number of Chinese population included in these randomized controlled studies is limited. Researches published in China are also non-systematic, therefore the included data is limited, leading to deviations in the results. Especially the conversion rate of different disease states, which is most likely to be affected. Second, in China, the out-of-pocket expenses in the medical process vary significantly among different groups of people. Some people pay the full amount at their own expense, some only pay a small part (paid by medical insurance), and some do not need to pay at all. This social phenomenon may lead to large differences in the choice of therapeutic drugs among different groups of people. Third, although we have included the cost of patients’ medical treatment, examinations, and medicines into the cost part, the cost of caring for patients, and salary loss were not included. Therefore, the disease may underestimate the quality of life of patients.

## Conclusions

In the Chinese population, oral anticoagulants are used to prevent AF-related cardiac embolism. Although warfarin treatment is less expensive, rivaroxaban is a better cost-effectiveness choice.

## Data Availability

The datasets generated and/or analysed during the current study are available upon request.

## Abbreviations

AF: Atrial fibrillation
QALYs: quality-adjusted life-years
ICER: incremental cost-effectiveness ratio
NOACs: Novel oral anticoagulants
NVAF: Non-valvular atrial fibrillation
IS: Ischemic stroke
ICH: Intracranial hemorrhage
ECH: Extracranial hemorrhage
MI: Myocardial infarction
Min: Minor
Mod: Moderate
Sev: Severe
Die: Dead
PwarST: Risk of cerebral infarction with warfarin
PwarICH: Risk of intracranial hemorrhage with warfarin
Umin: Quality of life adjusted years in patients with mild dysfunction
GDP: Gross domestic product
